# Association of clear vs blue-light filtering intraocular lenses with mental and behavioral disorders and diseases of the nervous system among patients receiving bilateral cataract surgery

**DOI:** 10.1097/j.jcrs.0000000000001184

**Published:** 2023-03-14

**Authors:** Minna Karesvuo, Piotr Kanclerz, Idan Hecht, Asaf Achiron, Raimo Tuuminen

**Affiliations:** From the Helsinki Retina Research Group, University of Helsinki, Helsinki, Finland (Karesvuo, Kanclerz, Hecht, Tuuminen); Health Services Dental Care, City of Helsinki, Helsinki, Finland (Karesvuo); Hygeia Clinic, Gdańsk, Poland (Kanclerz); Department of Ophthalmology, Shamir Medical Center, Tel Aviv, Israel (Hecht); Sackler School of Medicine, Tel Aviv University, Tel Aviv, Israel (Hecht, Achiron); Tel Aviv Sourasky Medical Center, Tel Aviv, Israel (Achiron); Department of Ophthalmology, Kymenlaakso Central Hospital, Kotka, Finland (Tuuminen).

## Abstract

To analyze new-onset mental and behavioral disorders and nervous system diseases in patients with cataract implanted with either non–blue-light filtering (BLF) or BLF intraocular lenses (IOLs) in both eyes.

**Setting:**

Department of Ophthalmology, Kymenlaakso Central Hospital, Kotka, Finland.

**Design:**

A retrospective registry-based cohort study of patients operated between September 2007 and December 2018 who were followed until December 2021. We included 4986 patients who underwent bilateral cataract surgery.

**Methods:**

Patients were implanted with either non-BLF IOLs (N = 2609) or BLF IOLs (N = 2377) in both eyes. Follow-up before the first-eye surgery and between the first-eye and the second-eye surgery was performed to acknowledge the preexisting disorders and diseases. After the second-eye surgery, the groups were analyzed for the new-onset mental and behavioral disorders and diseases of the nervous system subcategorized by the International Classification of Diseases codes.

**Results:**

1707 male and 3279 female patients, aged 73.2 ± 8.6 years at the first-eye surgery and 74.3 ± 8.8 years at the second-eye surgery, were identified. In univariate log-rank tests, the use of BLF IOLs showed no association in overall new-onset disorders or diseases over non-BLF IOLs, in any subtype diagnosis codes except for sleep disorders, which favored BLF IOLs (*P* = .003). A multivariate analysis adjusted for age and sex identified no associations in any new-onset disorders or diseases. Multivariate analysis of sleep disorders showed a nonsignificant advantage for BLF-IOLs (hazard ratio 0.756, 95% CI 0.534-1.070, *P* = .114).

**Conclusions:**

BLF IOLs were not associated with mental and behavioral disorders or diseases of the nervous system.

## Abstract 2

No associations of new-onset diagnoses were found for the use of blue-light filtering IOLs for health and neurological diseases after cataract surgery.

In a healthy human lens, the total visible light transmission decreases with age, which is more prominent for short wavelengths.^1^ Although intraocular lenses (IOLs) absorb UV light, only blue-light filtering (BLF) IOLs have a chromophore that absorbs short-wavelength light. BLF IOLs were designed to approximate the natural, healthy crystalline lens's light filtration and to protect the retina from high-energy wavelengths of the blue-light spectrum.^2^ Animal studies have shown that short-wavelengths can potentially lead to phototoxic retinal damage.^3^ In vitro studies of human retinal pigment epithelium (RPE) cells demonstrated that blue-light exposure initiates apoptosis of RPE cells, whereas BLF IOLs reduce this process.^4^ Protection from the blue-light spectrum might be particularly important among patients with vision impairment because of age-related macular degeneration (AMD), and there are clinical studies suggesting a link between blue-light exposure and the development of AMD. In the Beaver Dam Eye Study, outdoor activity and sun exposure early in life were associated with the development of AMD later in life.^5^ A similar observation by Taylor et al. showed that patients who had significantly higher exposure to blue or visible light over the preceding 20 years were more likely to have advanced types of AMDs such as geographic atrophy or disciform scarring.^6^

Blue-light is only partially responsible for photopic vision but is critical for scotopic vision.^7^ The decrease in scotopic visual function naturally occurring with age contributes to the increased risk of falling and for difficulties in driving and reading road signs at night in the elderly.^8,9^ Although BLF IOLs provide similar or better postoperative contrast sensitivity at select frequencies when compared with non-BLF IOLs, there are concerns that BLF IOLs might impair the ability to see in the dark.^10,11^ Furthermore, straylight, the cause of disability glare is increased in pseudophakes with BLF IOLs when compared with non-BLF IOLs.^12^ Our previous study has shown that BLF IOLs were not associated with reduced risk of injuries or driving accidents, whereas glare during nighttime driving was significantly worse in the BLF IOL group with pseudophakia.^13^ Blue-light can also negatively affect retinal ganglion cell function which is particularly important in glaucoma.^14,15^

Previous studies have suggested that phacoemulsification cataract surgery with implantation of clear (non-BLF) IOLs may lead to improvement in depressive symptoms and the number of health contacts for depression and/or anxiety.^16^ The photosensitive retinal ganglion cells, which are hypothesized to be responsible for behavioral and physiological functions separate from sight, are particularly sensitive to blue-light.^17,18^ As blue-light influences cognitive performance and seems necessary for good mental and physical health, permanent blue-light reduction might have negative influences on mental health and on optimal vision in low light, but the topic remains controversial.^19–21^ The aim of this study was to analyze mental and behavioral disorders and nervous system diseases in patients with cataract implanted with either non-BLF or BLF IOLs in both eyes.

## METHODS

### Study Design

Mental and behavioral disorders (International Classification of Diseases, Tenth Revision [ICD-10] F00-F09) and nervous system diseases (ICD-10 G00-G09) were studied in patients with cataract implanted with either non-BLF or BLF IOLs in both eyes. Analyses of subgroups of these conditions were based on the ICD-10 coding. Furthermore, specific ICD-10 codes based on previous studies were focused in subanalyses.

For every cataract surgery patient in the registry, all diagnosis codes for mental and behavioral disorders and for nervous system diseases were retrieved from the year 2007 from the patient medical records. Diagnoses for mental and behavioral disorders and for nervous system diseases which were registered before the first-eye surgery or between the first-eye and the second-eye surgery were acknowledged in the analyses, and these specific diagnoses were excluded from being new-onset diseases if they were observed also after the second-eye surgery.

The study was approved by the Research Director and the Chief Medical Officer of the Kymenlaakso Central Hospital, Kotka, Finland, and adhered to the tenets of the Declaration of Helsinki. This was a retrospective registry-based cohort study of consecutive cataract surgeries performed between 2007 and 2018 at the Department of Ophthalmology of the Kymenlaakso Central Hospital. This report follows the STROBE reporting guidelines for observational studies.^22^

### Patients

All adult patients (aged at least 18 years) who underwent uneventful phacoemulsification surgery and in-the-bag implantation of a non-BLF IOL (ZA9003 and ZCB00/PCB00, Tecnis, Abbott Medical Optics, Inc. and Johnson & Johnson Vision) or a BLF IOL (SN60WF/AU00T0, Acrysof, Alcon Laboratories, Inc.) to both eyes were included in the study.

The Department of Ophthalmology is a governmental tax-financed Unit covering the Hospital District of approximately 175 000 inhabitants. All referred cataract surgery patients are allocated to the treating physicians from the cataract surgery operation queue solely by the hospital. The IOL type was assigned at the discretion of the operating surgeon and was not randomly allocated. The patient had no choice in the types of lenses implanted in their eyes. There were no financial arguments in selecting between the non-BLF and BLF IOLs. The clinical practice of the Unit was to use the same IOL type for the contralateral eye. There were no other guidelines given in the Unit as to which IOL type should be chosen in any situation. Both IOL types (non-BLF and BLF) were used constantly and throughout the study period. Both IOL types are made of acrylic and are of similar design, making them proper candidates to assess the associations of the blue-light filtering aspect, which is the key difference between them. Before surgery, all patients underwent a complete ophthalmological examination including corrected distance visual acuity (CDVA) evaluation, tonometry, and slitlamp examination, including anterior segment and fundoscopy assessment, as well as biometry.

### Mental Health and Neurological Diseases

ICD-10 coding was used to specify disease subtypes. These were as follows: F00-F09—Mental disorders due to known physiological conditions; F10-F19—Mental and behavioral disorders due to psychoactive substance use; F20-F29—Schizophrenia, schizotypal, delusional, and other nonmood psychotic disorders; F30-F39—Mood (affective) disorders; F40-F49—Anxiety, dissociative, stress-related, somatoform, and other nonpsychotic mental disorders; F50-F59—Behavioral syndromes associated with physiological disturbances and physical factors; F60-F69—Disorders of adult personality and behavior; F70-F79—Intellectual disabilities; F80-F89—Pervasive and specific developmental disorders; F99—Unspecified mental disorder; G00-G09—Inflammatory diseases of the central nervous system; G10-G13—Systemic atrophies primarily affecting the central nervous system; G20-G26—Extrapyramidal and movement disorders; G30-G32—Other degenerative diseases of the nervous system; G35-G37—Demyelinating diseases of the central nervous system; G40-G47—Episodic and paroxysmal disorders of shoulder and upper arm; G50-G59—Nerve, nerve root, and plexus disorders; G60-G64—Polyneuropathies and other disorders of the peripheral nervous system; G70-G73—Diseases of myoneural junction and muscle; G80-G83—Cerebral palsy and other paralytic syndromes; G90-G99—Other disorders of the nervous system. The types of diagnoses were obtained from the patient medical records.

### Cataract Surgery

According to the Finnish National Guidelines for cataract operations, the CDVA for cataract surgery is recommended to be 0.5/0.3 in the better/worse eye or less by Snellen equivalents, except under some specific circumstances (Current Care Guidelines for Cataracts). The surgical technique used in this study was phacoemulsification (Infinity/Centurion Vision System, Alcon Laboratories, Inc.) with a 2.4 to 2.75 mm clear corneal incision.

### Death Reported and Censoring

When a death certificate is issued, a copy goes to the population register (The Digital and Population Data Services Agency, Finland). As a rule, the date of death is published in a daily update, which is uploaded weekly on Thursdays to the patient medical registry (Lifecare, Tietoevry, Espoo, Finland). In this update, the data on death cover all patients of a Hospital District. When death is reported in the daily data, the Lifecare patient medical record program automatically deletes postdeath appointments and closes referrals. Death was used as a censoring event to improve follow-up precision.

### Statistics

Unless otherwise specified, data are presented as mean ± SD. In case the diagnosis of mental disorders or neurological diseases was found in the patient medical records preceding the first-eye surgery or between the first-eye and second-eye surgeries, the case was excluded from the specific analysis. Only the first diagnosis after the second-eye surgery was recorded. For survival analyses, the follow-up time was counted to the first event or when censoring the data. Patients were censored when death or the end of follow-up was reached. Kaplan-Meier curves were generated, and multivariate Cox regression controlling for age and sex was used to estimate hazard ratios (HRs). Statistical analysis was performed using IBM SPSS Statistics 27 (IBM Corp.). The *P* value of less than 0.05 was considered statistically significant.

## RESULTS

Included were 4986 patients (9972 eyes) who underwent bilaterally uneventful cataract surgery; 1707 male and 3279 female patients, aged 74.5 ± 8.8 years at the first-eye surgery and 75.7 ± 8.8 years at the second-eye surgery. In total, 2377 patients (4754 eyes) were operated on with BLF IOLs and 2609 patients (5218 eyes) with non-BLF IOLs. The mean follow-up time was 4.9 ± 2.8 years preceding the first-eye surgery and 5.9 ± 3.0 years after the second-eye surgery (Table 1).

**Table 1.** Baseline variables between the BLF and non-BLF IOL types

Parameter	BLF IOL (N = 2377)	Non-BLF IOL (N = 2609)	*P* value
M:F (n/%)	729:1648 (30.7:69.3%)	978:1631 (37.5:62.5%)	<.001^b^
Age at first-eye surgery (y)	76.6 ± 7.8	72.7 ± 9.3	<.001^c^
Age at second-eye surgery (y)	77.9 ± 7.8	73.7 ± 9.2	<.001^c^
Follow-up before first-eye surgery (y)^a^	4.4 ± 2.8	5.4 ± 2.7	<.001^c^
Follow-up after second-eye surgery (y)	6.0 ± 3.2	5.9 ± 2.9	.153

BLF = blue-light filtering

Data are given as mean ± SD or absolute numbers with proportions. Patients operated on both eyes with either non-BLF IOLs (2609 patients; 5218 eyes) or BLF IOLs (2377 patients; 4754 eyes) were compared.

a Follow-up before the first-eye surgery to acknowledge the preexisting disorders and diseases

For 2-group comparisons, ^b^qualitative data were analyzed with the 2-factor chi-squared test and ^c^continuous variables with the *t* test

Associations were analyzed between different IOL types and new-onset mental and behavioral disorders and diseases of the nervous system. After the second-eye surgery, the total number of patients with any new-onset F00-F99 diagnoses for the category of “mental, behavioral, and neurodevelopmental disorders” existed in 353 patients (N = 160 for BLF and N = 193 for non-BLF groups) and with any new-onset G00-G99 diagnoses for the category of “diseases of the nervous system” existed in 1071 patients (N = 547 for BLF and N = 524 for non-BLF groups) (Tables 2 and 3).

**Table 2.** Age-adjusted and sex-adjusted hazard ratios of new-onset mental, behavioral, and neurodevelopmental disorders between the IOL types according to ICD-10 classification

Diagnoses	Cases, n	BLF vs non-BLF IOL	*P* value
F00-F09—Mental disorders due to known physiological conditions	145	0.857 (0.609-1.207)	.378
F10-F19—Mental and behavioral disorders due to psychoactive substance use	63	1.349 (0.741-2.455)	.327
F20-F29—Schizophrenia, schizotypal, delusional, and other nonmood psychotic disorders	29	0.751 (0.322-1.754)	.508
F30-F39—Mood (affective) disorders	96	1.116 (0.738-1.688)	.604
F40-F49—Anxiety, dissociative, stress-related, somatoform, and other nonpsychotic mental disorders	74	0.747 (0.462-1.210)	.236
F50-F59—Behavioral syndromes associated with physiological disturbances and physical factors	58	0.649 (0.373-1.130)	.126
F00-F99—Any new-onset F subcode	353	0.879 (0.699-1.104)	.267

BLF = blue-light filtering; HR = hazard ratio; ICD-10 = International Classification of Diseases, Tenth Revision

Data are given as adjusted HR with 95% CI for diagnoses with number of cases 5 or more. Patients were operated on both eyes with either non-BLF IOLs (2609 patients; 5218 eyes) or BLF IOLs (2377 patients; 4754 eyes). F60-F69 (N = 2), F70-F79 (N = 3), F80-F89 (N = 3), F90-F98 (N = 0), and F99 (N = 1) were included in the “any new-onset F subcode” analysis.

**Table 3.** Age-adjusted and sex-adjusted hazard ratios of new-onset diseases of the nervous system between the IOL types according to ICD-10 classification

Diagnoses	Cases, n	BLF vs non-BLF IOL	*P* value
G20-G26—Extrapyramidal and movement disorders	100	0.820 (0.547-1.229)	.336
G30-G32—Other degenerative diseases of the nervous system	441	1.116 (0.918-1.356)	.270
G40-G47—Episodic and paroxysmal disorders shoulder and upper arm	376	0.927 (0.751-1.144)	.478
G50-G59—Nerve, nerve root, and plexus disorders	203	0.957 (0.719-1.272)	.124
G60-G64—Polyneuropathies and other disorders of the peripheral nervous system	68	1.275 (0.779-2.088)	.334
G70-G73—Diseases of myoneural junction and muscle	26	0.861 (0.384-1.929)	.716
G80-G83—Cerebral palsy and other paralytic syndromes	9	1.185 (0.302-4.646)	.787
G90-G99—Other disorders of the nervous system	17	2.203 (0.787-6.168)	.133
G00-G99—Any new-onset G subcode	1071	0.999 (0.883-1.130)	.985

BLF = blue-light-filtering; HR = hazard ratio; ICD-10 = International Classification of Diseases, Tenth Revision

Data are given as adjusted HR with 95% CI for diagnoses with number of cases 5 or more. Patients were operated on both eyes with either non-BLF IOLs (2609 patients; 5218 eyes) or BLF IOLs (2377 patients; 4754 eyes). G00-G09 (N = 2), G10-G13 (N = 3), and G35-G37 (N = 1) were included in the “any new-onset G subcode” analysis.

### Mental and Behavioral Disorders

Mental and behavioral disorders were most frequently related to mental disorders due to known physiological conditions (N = 145); mood (affective) disorders (N = 96); anxiety, dissociative, stress-related, somatoform, and other nonpsychotic mental disorders (N = 74); mental and behavioral disorders due to psychoactive substance use (N = 63); and behavioral syndromes associated with physiological disturbances and physical factors (N = 58) (Table 2).

**Figure 1.**
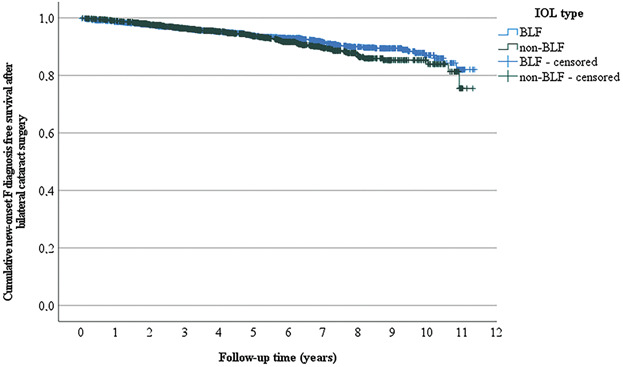
Kaplan-Meier plot of mental and behavioral disorder-free survival after the second-eye cataract surgery according to the type of IOL. In univariate analysis, mental and behavioral disorder-free survival after the second-eye surgery was comparable between patients with non-BLF and BLF IOLs (*P* = .205 in a log-rank [Mantel-Cox] test). BLF = blue-light filtering

In univariate analysis, mental and behavioral disorder-free survival after the second-eye surgery was comparable among patients with BLF IOLs compared with non-BLF IOLs (*P* = .205 with log-rank, Figure 1). In multivariate Cox regression analysis controlling for age and sex, the type of IOL (BLF vs non-BLF IOL) did affect neither the mental and behavioral disorder-free survival rate (HR 0.879, 95% CI 0.699-1.104, *P* = .267) nor any of the diagnosis subgroups (Table 2).

### Nervous System Diseases

Diseases of the nervous system were most frequently related to the following subgroups: other degenerative diseases of the nervous system (N = 441); episodic and paroxysmal disorders (N = 376); nerve, nerve root, and plexus disorders (N = 206); extrapyramidal and movement disorders (N = 100); and polyneuropathies and other disorders of the peripheral nervous system (N = 68) (Table 3).

**Figure 2.**
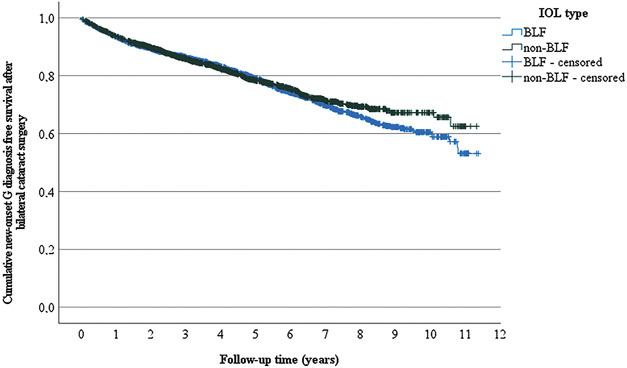
Kaplan-Meier plot of nervous system disease-free survival after the second-eye cataract surgery according to the type of IOL. In univariate analysis, nervous system disease-free survival after the second-eye surgery was comparable between patients with non-BLF and BLF IOLs (*P* = .342 in a log-rank [Mantel-Cox] test). BLF = blue-light filtering

In univariate analysis, nervous system disease-free survival after the second-eye surgery was comparable among patients with BLF IOLs compared with non-BLF IOLs (*P* = .342 with log-rank, Figure 2). In multivariate Cox regression analysis controlling for age and sex, the type of IOL (BLF vs non-BLF IOL) did affect neither the nervous system disease-free survival rate (HR 0.999, 95% CI 0.883-1.130, *P* = .985) nor any of the diagnosis subgroups (Table 3).

### Sleep Disorders

Next, specific diagnostic subcodes within mental and behavioral disorders and nervous system diseases were analyzed. A separate analysis of specific codes for F01 (vascular dementia), F02 (dementia in other diseases classified elsewhere), F03 (unspecified dementia), F20 (schizophrenia), F32 (major depressive disorder, single episode), F33 (major depressive disorder, recurrent), F41 (other anxiety disorders), G20 (Parkinson disease), G30 (Alzheimer disease), G40 (epilepsy and recurrent seizures), and G43 (migraine) showed no statistically significant differences between the BLF and non-BLF groups in multivariate analysis adjusted for age and sex (Table 4).

**Table 4.** Univariate and multivariate (age-adjusted and sex-adjusted) hazard ratios of new-onset specific subcodes in mental and behavioral disorders and diseases of the nervous system between the IOL types

Diagnoses	Cases, n (BLF:non-BLF)	BLF vs non-BLF IOL		
		Univariate	Multivariate	
		*P* value	HR (95% CI)	*P* value
F01-F03—Dementia	107 (60:47)	.271	0.917 (0.620, 1.356)	.663
F20—Schizophrenia	6 (1:5)	.105	0.237 (0.027, 2.106)	.197
F32-F33—Major depressive disorders	80 (38:42)	.558	1.025 (0.651, 1.614)	.915
F41—Other anxiety disorders	54 (21:33)	.071	0.720 (0.409, 1.266)	.254
F51—Sleep disorders^a^	56 (23:33)	.167	0.662 (0.382, 1.148)	.142
G20—Parkinson disease	60 (34:26)	.255	1.212 (0.716, 2.050)	.473
G30—Alzheimer disease	431 (259:172)	<.001	1.109 (0.911, 1.351)	.303
G40—Epilepsy and recurrent seizures	60 (34:26)	.236	1.526 (0.898, 2.593)	.118
G43—Migraine	9 (1:8)	.021	0.153 (0.019, 1.255)	.080
G47—Sleep disorders	145 (54:91)	.003	0.756 (0.534, 1.070)	.114

BLF = blue-light filtering; HR = hazard ratio; ICD-10 = International Classification of Diseases, Tenth Revision

Data are given as adjusted HR with 95% CI for diagnoses with number of cases 5 or more. Patients were operated on both eyes with either non-BLF IOLs (2609 patients; 5218 eyes) or BLF IOLs (2377 patients; 4754 eyes).

a F51—Sleep disorders not due to a substance or known physiological condition

Furthermore, new-onset sleep disorders after the second-eye surgery were analyzed with the following specific diagnosis subcodes: F51 (sleep disorders not due to a substance or known physiological condition) and G47 (sleep disorders). In total, we found 56 patients with a new-onset F51 subcode and 147 patients with a new-onset G47 subcode. In univariate analysis, there were no significant differences for F51; sleep disorders not due to a substance or known physiological condition (*P* = .167 with log-rank), but significant benefit favoring BLF IOLs over non-BLF IOLs for G47; sleep disorders (*P* = .003 with log-rank) (Supplement Figures 1 and 2, available at http://links.lww.com/JRS/A845 and http://links.lww.com/JRS/A846). In multivariate Cox regression analysis controlling for age and sex, the type of IOL (BLF vs non-BLF IOLs) showed non-significant benefit favoring BLF IOLs over non-BLF IOLs for F51; sleep disorders not due to a substance or known physiological condition (HR 0.662, 95% CI 0.382-1.148, *P* =.142), and for G47; sleep disorders (HR 0.756, 95% CI 0.534-1.070, *P* =.114) (Supplement Figures 3 and 4, available at http://links.lww.com/JRS/A847 and http://links.lww.com/JRS/A848).

## DISCUSSION

In this large group of individuals undergoing cataract surgery, nearly half received BLF IOLs, while the other had clear IOLs implanted. Mental and neurological diseases over a long follow-up period after bilateral cataract surgery were assessed using the medical coding system. During the mean follow-up period of over 5 years, new-onset mental disorder and neurological disease-free survival were not different between patients with BLF and non-BLF IOLs.

Increasing retinal light exposure by phacoemulsification cataract surgery has been shown to alleviate symptoms of depression and the number of required health contacts for depression and anxiety.^16^ Moreover, it has been hypothesized that blue-light in particular is important for good mental and physical health, and permanent blue-light reduction might have negative influences on mental health.^20^ BLF IOLs have been widely used in clinical practice for more than 20 years and have been implanted in the eyes of millions of patients worldwide. Still, there is very little evidence on the influence of BLF IOLs on mental health and nervous system disorders, and the studies that have been published reported conflicting results. Zambrowski et al. found no difference between BLF and non-BLF IOLs in change of mood state, and the depression index 8 weeks after cataract surgery.^23^ Consistent with these results, Schmoll et al. reported improvement in reaction time and reduction in daytime sleepiness after first-eye cataract surgery, but not after second-eye surgery; the choice of BLF or non-BLF IOL made no difference in the magnitude of cognitive impairment.^24^ However, in contrast with these studies, in the study by Chellappa et al., cognitive function was indexed by tasked tapping onto attentional resources (psychomotor vigilance task slowest reaction times).^25^ Patients with non-BLF IOLs had faster reaction times during light exposure, and greater percentage of correct answers compared with patients with BLF IOLs.^25^ Ayaki et al. noted that the Pittsburg Sleep Quality Index showed greater improvement in sleep latency and sleep disturbance in patients receiving non-BLF than BLF IOL.^26^ However, the subjective systemic health analyzed in the National Eye Institute Visual Function Questionnaire was not different between BLF and non-BLF IOLs. Finally, Griepentrog et al. found no difference in the risk of developing dementia between BLF and non-BLF IOLs.^27^ However, BLF IOLs increased the risk of death compared with non-BLF IOLs which potentially is associated with perturbation of circadian biology by BLF IOLs.^27^ The previously conducted investigations had a modest sample size and were relatively selective because they did not analyze a wide spectrum of mental and behavioral disorders and nervous system diseases. They also commonly included only unilateral cataract surgery, which permits blue-light stimulation to exist from the contralateral eye, weakening the study design.

The effect of BLF IOLs on sleep specifically has been the subject of some debate.^28^ Evidence shows that the quality of sleep and sleep latency can also be affected by cataract extraction. It has been suggested that filtration of blue-light might interfere with the normal circadian rhythm and sleep, although conflicting evidence exists. Relatively few studies have examined BLF IOLs in this context until now. Brøndsted et al. reported that BLF IOLs increase sleep efficiency, but lower nocturnal melatonin secretion compared with non-BLF IOLs.^28^ It is interesting that in the results seen here, of the many mental and neurological health outcomes, sleep disorders (except sleep disorders not due to a substance or known physiological condition) were the only disorder influenced by the choice of IOL, favoring BLF IOLs. When adjusting for confounders, however, the difference loses significance. Nonetheless, this is an interesting result and encourages further investigation.

Our investigation has limitations. First, allocation to the IOL type was not randomized but on the operating surgeon's discretion and the contralateral eye lens status. Certain differences could therefore exist between groups. For instance, the prevalence of dementia is higher in women than in men and is known to double in about every 5 years until age 85 years.^29^ Furthermore, BLF-IOLs compared with non-BLF IOLs may have been more frequently implanted in elderly patients due to the blue-light filtration properties of the IOL and suspected protection against age-related macular degeneration, but this could not be ascertained. To mitigate this, multivariate analysis was performed to eliminate the influence of demographic parameters such as age and sex. Second, we did not investigate worsening of preexisting disorders because this was outside the scope of this investigation. Our study focused only on new-onset diseases and preexisting diseases before the first-eye surgery or between the first-eye and second-eye surgeries were acknowledged in case the specific diseases existed in the medical records between the start of data retrieve from year 2007 and the second-eye surgery. Furthermore, one should keep in mind that the study took place in Finland, where light levels in winter months are low, so outcomes from other geographical latitudes could potentially be different. All the patients were treated in a single center with similar equipment and criteria for surgery, but other covariates beyond age and sex influencing the operating surgeon's decision on the type of IOL (BFL vs non-BLF) could not be controlled. For instance, the Kaplan-Meier curves and Cox survival analyses did not assess whether the participants had a familial predisposition for a disease or were suffering from an undiagnosed disorder. These aspects, however, are estimated to have little influence on the surgeon's choice of IOL. Some of the subgroups may seem irrelevant regarding the IOL properties, but all the subgroup diagnoses were included in the analysis of any new-onset F or G subcode to analyze the overall morbidity for mental disorders and neurological diseases. The subgrouping was based on the ICD-10 coding, which was used in daily clinical practice, any other kind of subgrouping of the diseases may have resulted in different outcomes. Finally, the study might have inherited bias associated with medical coding (eg, unintentional misspecification, unbundling, and upcoding may lead to coding inaccuracy); still, these aspects are estimated to influence both groups similarly. The Finnish registry is considered highly accurate, and we intended to receive and operate the highest quality and standardized data.^30^ The strength of our study is that we had access to the patient medical records for every individual in the study, our database covers a large population and comprehensively captures all hospitalizations and diagnoses after surgery.

Increasing retinal light exposure by phacoemulsification cataract surgery has been shown to alleviate mental health conditions and nervous system disorders; studies analyzing the influence of BLF IOLs reported conflicting results. In this large cohort study of patients who underwent uneventful cataract surgery to both eyes, BLF IOLs were not associated with overall new-onset mental disorders or diseases of the nervous system over the non-BLF IOLs. These findings could broaden our understanding of the potential advantages and disadvantages of short-wavelength filtration of IOLs.

**WHAT WAS KNOWN**

- Blue-light filtering (BLF) IOLs were designed to protect against age-related macular degeneration. Short-wavelength light may affect photosensitive retinal ganglion cells and affect mental health and diseases of the nervous system.

**WHAT THIS PAPER ADDS**

- The use of BLF IOLs did not promote new-onset mental and behavioral disorders and nervous system diseases after the bilateral cataract surgery.
